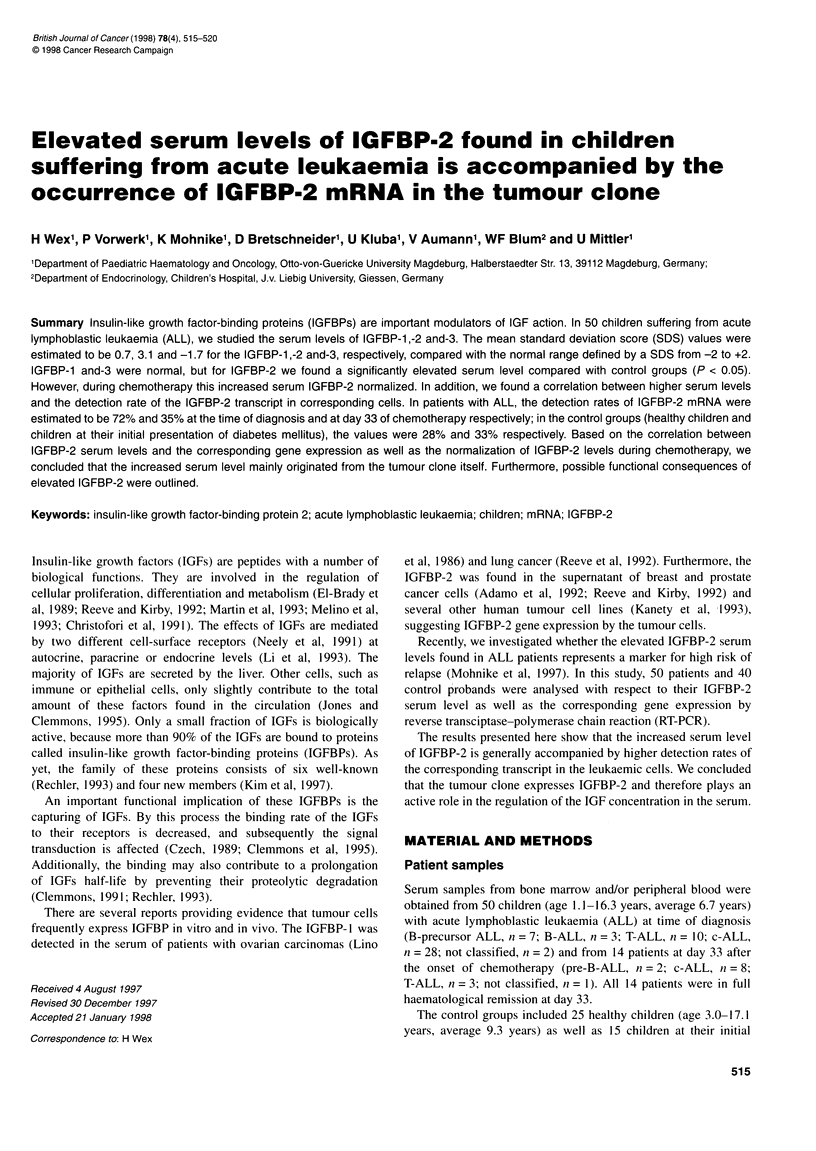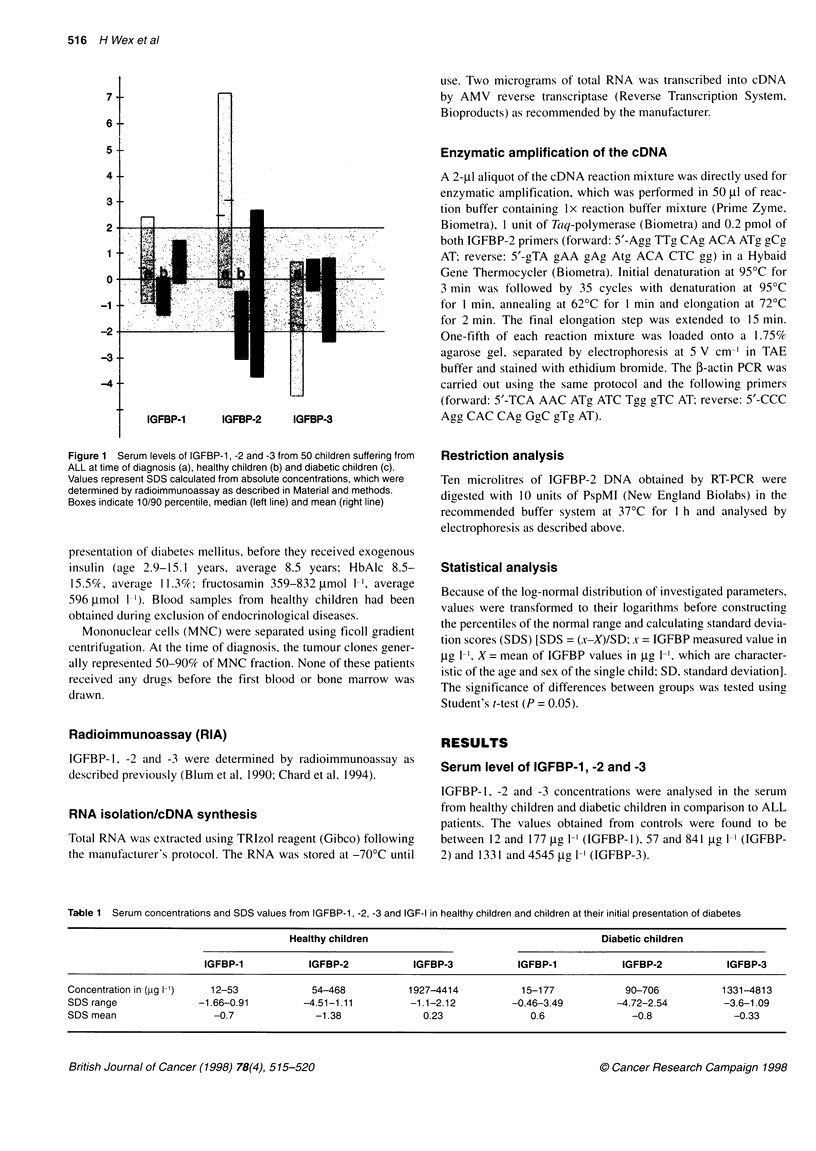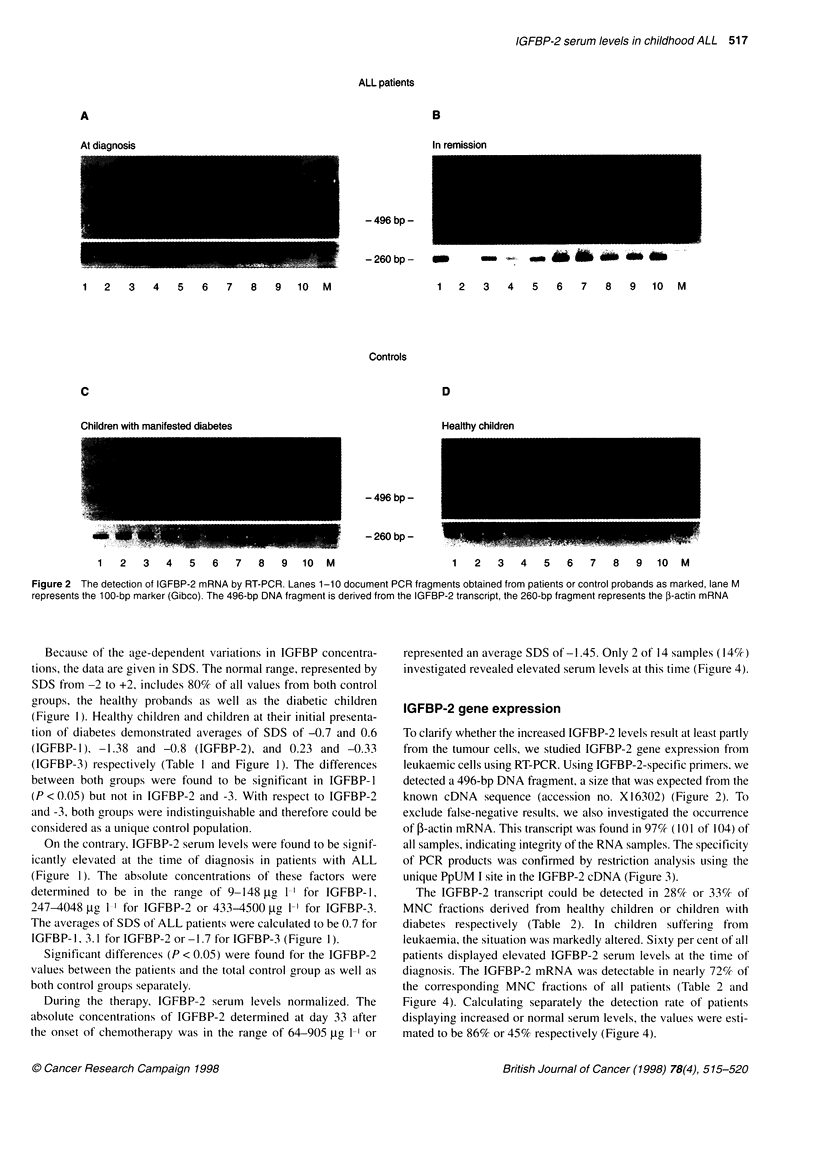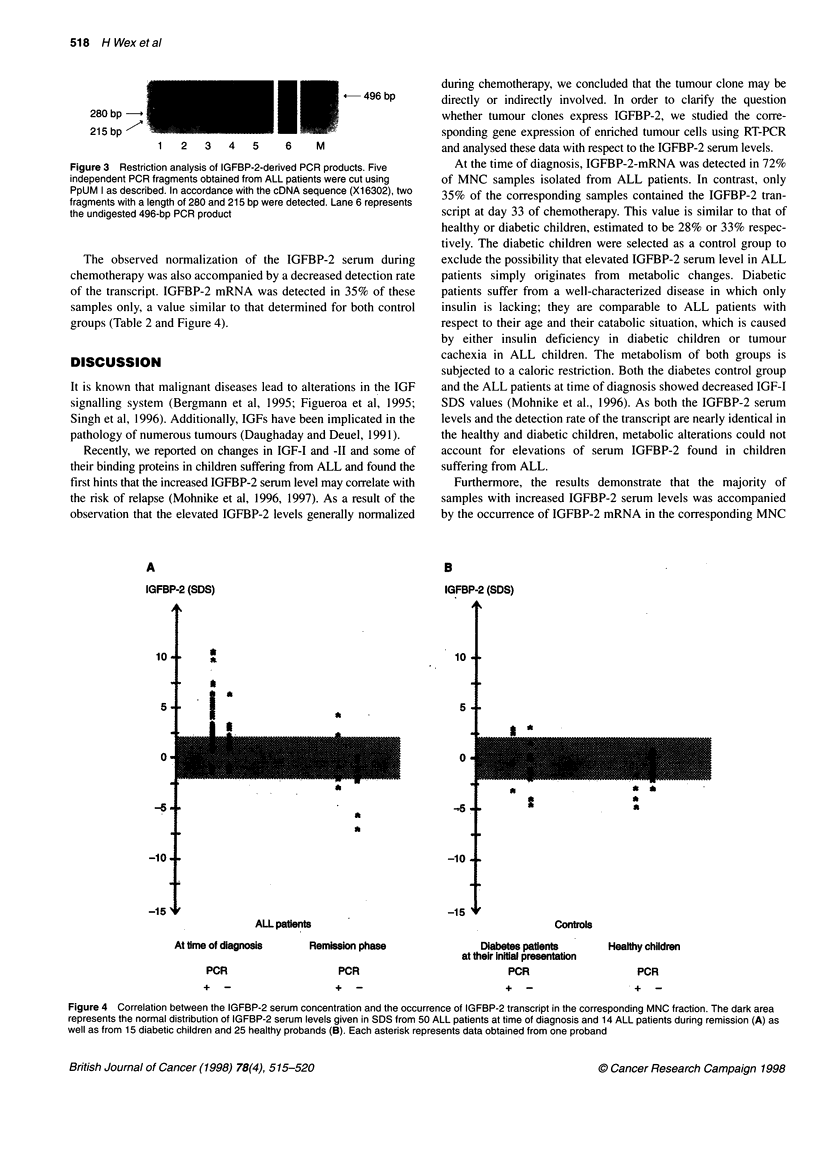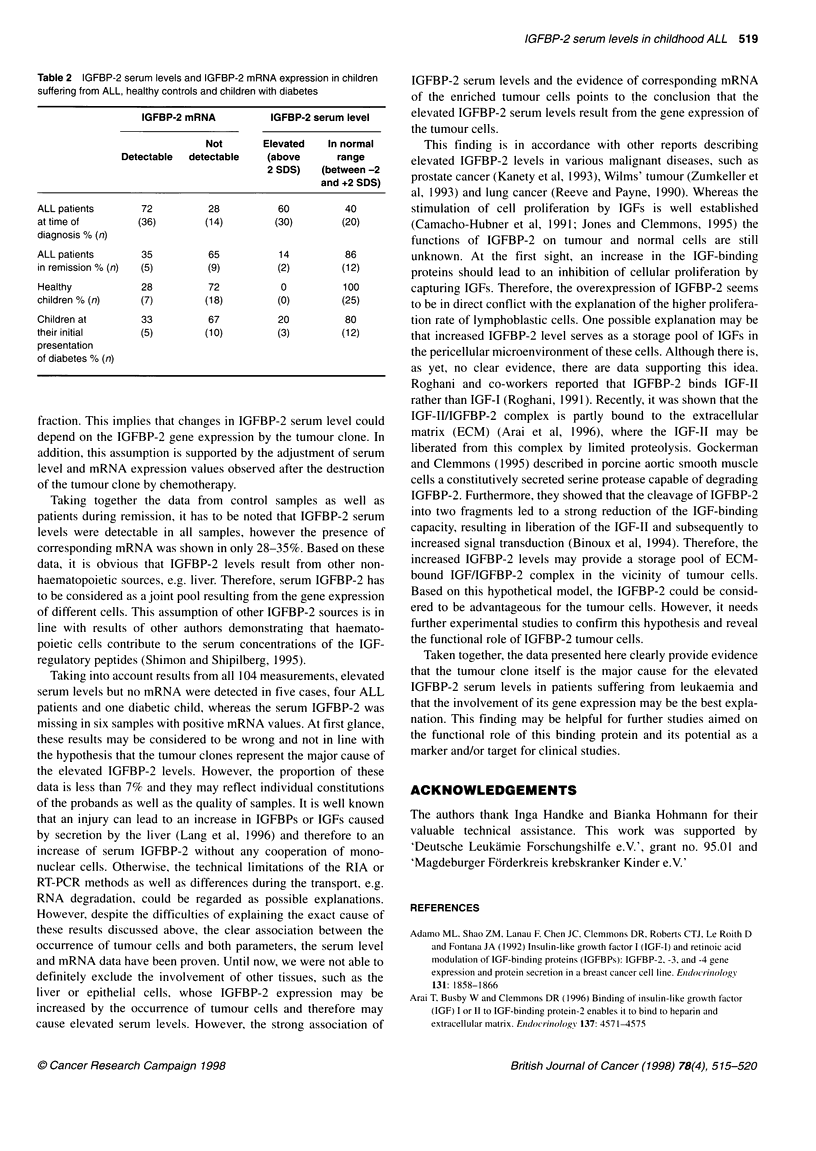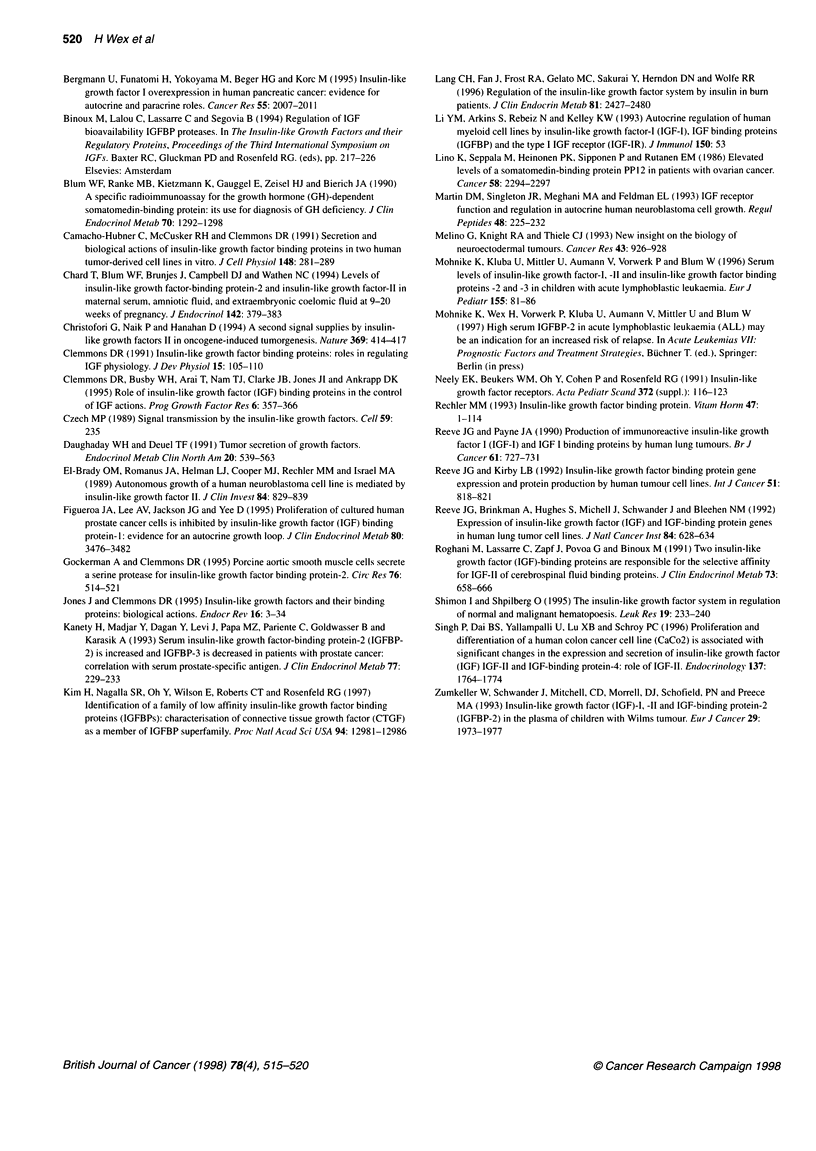# Elevated serum levels of IGFBP-2 found in children suffering from acute leukaemia is accompanied by the occurrence of IGFBP-2 mRNA in the tumour clone.

**DOI:** 10.1038/bjc.1998.525

**Published:** 1998-08

**Authors:** H. Wex, P. Vorwerk, K. Mohnike, D. Bretschneider, U. Kluba, V. Aumann, W. F. Blum, U. Mittler

**Affiliations:** Department of Paediatric Haematology and Oncology, Otto-von-Guericke University Magdeburg, Germany.

## Abstract

**Images:**


British Journal of Cancer (1998) 78(4), 515–520
© 1998 Cancer Research Campaign

### Elevated serum levels of IGFBP-2 found in children suffering from acute leukaemia is accompanied by the occurrence of IGFBP-2 mRNA in the tumour clone

**H Wex[1], P Vorwerk[1], K Mohnike[1], D Bretschneider[1], U Kluba[1], V Aumann[1], WF Blum[2] and U Mittler[1]**

[1]Department of Paediatric Haematology and Oncology, Otto-von-Guericke University Magdeburg, Halberstaedter Str. 13, 39112 Magdeburg, Germany; [2]Department of Endocrinology, Children's Hospital, J.v. Liebig University, Giessen, Germany

**Summary** Insulin-like growth factor-binding proteins (IGFBPs) are important modulators of IGF action. In 50 children suffering from acute lymphoblastic leukaemia (ALL), we studied the serum levels of IGFBP-1,-2 and-3. The mean standard deviation score (SDS) values were estimated to be 0.7, 3.1 and –1.7 for the IGFBP-1,-2 and-3, respectively, compared with the normal range defined by a SDS from –2 to +2. IGFBP-1 and-3 were normal, but for IGFBP-2 we found a significantly elevated serum level compared with control groups ($P < 0.05$). However, during chemotherapy this increased serum IGFBP-2 normalized. In addition, we found a correlation between higher serum levels and the detection rate of the IGFBP-2 transcript in corresponding cells. In patients with ALL, the detection rates of IGFBP-2 mRNA were estimated to be 72% and 35% at the time of diagnosis and at day 33 of chemotherapy respectively; in the control groups (healthy children and children at their initial presentation of diabetes mellitus), the values were 28% and 33% respectively. Based on the correlation between IGFBP-2 serum levels and the corresponding gene expression as well as the normalization of IGFBP-2 levels during chemotherapy, we concluded that the increased serum level mainly originated from the tumour clone itself. Furthermore, possible functional consequences of elevated IGFBP-2 were outlined.

**Keywords:** insulin-like growth factor-binding protein 2; acute lymphoblastic leukaemia; children; mRNA; IGFBP-2

Insulin-like growth factors (IGFs) are peptides with a number of biological functions. They are involved in the regulation of cellular proliferation, differentiation and metabolism (El-Brady et al, 1989; Reeve and Kirby, 1992; Martin et al, 1993; Melino et al, 1993; Christofori et al, 1991). The effects of IGFs are mediated by two different cell-surface receptors (Neely et al, 1991) at autocrine, paracrine or endocrine levels (Li et al, 1993). The majority of IGFs are secreted by the liver. Other cells, such as immune or epithelial cells, only slightly contribute to the total amount of these factors found in the circulation (Jones and Clemmons, 1995). Only a small fraction of IGFs is biologically active, because more than 90% of the IGFs are bound to proteins called insulin-like growth factor-binding proteins (IGFBPs). As yet, the family of these proteins consists of six well-known (Rechler, 1993) and four new members (Kim et al, 1997).

An important functional implication of these IGFBPs is the capturing of IGFs. By this process the binding rate of the IGFs to their receptors is decreased, and subsequently the signal transduction is affected (Czech, 1989; Clemmons et al, 1995). Additionally, the binding may also contribute to a prolongation of IGFs half-life by preventing their proteolytic degradation (Clemmons, 1991; Rechler, 1993).

There are several reports providing evidence that tumour cells frequently express IGFBP in vitro and in vivo. The IGFBP-1 was detected in the serum of patients with ovarian carcinomas (Lino et al, 1986) and lung cancer (Reeve et al, 1992). Furthermore, the IGFBP-2 was found in the supernatant of breast and prostate cancer cells (Adamo et al, 1992; Reeve and Kirby, 1992) and several other human tumour cell lines (Kanety et al, 1993), suggesting IGFBP-2 gene expression by the tumour cells.

Recently, we investigated whether the elevated IGFBP-2 serum levels found in ALL patients represents a marker for high risk of relapse (Mohnike et al, 1997). In this study, 50 patients and 40 control probands were analysed with respect to their IGFBP-2 serum level as well as the corresponding gene expression by reverse transciptase–polymerase chain reaction (RT-PCR).

The results presented here show that the increased serum level of IGFBP-2 is generally accompanied by higher detection rates of the corresponding transcript in the leukaemic cells. We concluded that the tumour clone expresses IGFBP-2 and therefore plays an active role in the regulation of the IGF concentration in the serum.

### MATERIAL AND METHODS

### Patient samples

Serum samples from bone marrow and/or peripheral blood were obtained from 50 children (age 1.1–16.3 years, average 6.7 years) with acute lymphoblastic leukaemia (ALL) at time of diagnosis (B-precursor ALL, $n = 7$; B-ALL, $n = 3$; T-ALL, $n = 10$; c-ALL, $n = 28$; not classified, $n = 2$) and from 14 patients at day 33 after the onset of chemotherapy (pre-B-ALL, $n = 2$; c-ALL, $n = 8$; T-ALL, $n = 3$; not classified, $n = 1$). All 14 patients were in full haematological remission at day 33.

The control groups included 25 healthy children (age 3.0–17.1 years, average 9.3 years) as well as 15 children at their initial

*Received 4 August 1997*
*Revised 30 December 1997*
*Accepted 21 January 1998*

*Correspondence to*: H Wex

**Figure 1** Serum levels of IGFBP-1, -2 and -3 from 50 children suffering from ALL at time of diagnosis (a), healthy children (b) and diabetic children (c). Values represent SDS calculated from absolute concentrations, which were determined by radioimmunoassay as described in Material and methods. Boxes indicate 10/90 percentile, median (left line) and mean (right line)

presentation of diabetes mellitus, before they received exogenous insulin (age 2.9–15.1 years, average 8.5 years; HbAlc 8.5–15.5%, average 11.3%; fructosamin 359–832 μmol $l^{-1}$, average 596 μmol $l^{-1}$). Blood samples from healthy children had been obtained during exclusion of endocrinological diseases.

Mononuclear cells (MNC) were separated using ficoll gradient centrifugation. At the time of diagnosis, the tumour clones generally represented 50–90% of MNC fraction. None of these patients received any drugs before the first blood or bone marrow was drawn.

### Radioimmunoassay (RIA)

IGFBP-1, -2 and -3 were determined by radioimmunoassay as described previously (Blum et al, 1990; Chard et al, 1994).

### RNA isolation/cDNA synthesis

Total RNA was extracted using TRIzol reagent (Gibco) following the manufacturer's protocol. The RNA was stored at −70°C until use. Two micrograms of total RNA was transcribed into cDNA by AMV reverse transcriptase (Reverse Transcription System, Bioproducts) as recommended by the manufacturer.

### Enzymatic amplification of the cDNA

A 2-μl aliquot of the cDNA reaction mixture was directly used for enzymatic amplification, which was performed in 50 μl of reaction buffer containing 1× reaction buffer mixture (Prime Zyme, Biometra), 1 unit of *Taq*-polymerase (Biometra) and 0.2 pmol of both IGFBP-2 primers (forward: 5′-Agg TTg CAg ACA ATg gCg AT; reverse: 5′-gTA gAA gAg Atg ACA CTC gg) in a Hybaid Gene Thermocycler (Biometra). Initial denaturation at 95°C for 3 min was followed by 35 cycles with denaturation at 95°C for 1 min, annealing at 62°C for 1 min and elongation at 72°C for 2 min. The final elongation step was extended to 15 min. One-fifth of each reaction mixture was loaded onto a 1.75% agarose gel, separated by electrophoresis at 5 V $cm^{-1}$ in TAE buffer and stained with ethidium bromide. The β-actin PCR was carried out using the same protocol and the following primers (forward: 5′-TCA AAC ATg ATC Tgg gTC AT; reverse: 5′-CCC Agg CAC CAg GgC gTg AT).

### Restriction analysis

Ten microlitres of IGFBP-2 DNA obtained by RT-PCR were digested with 10 units of PspMI (New England Biolabs) in the recommended buffer system at 37°C for 1 h and analysed by electrophoresis as described above.

### Statistical analysis

Because of the log-normal distribution of investigated parameters, values were transformed to their logarithms before constructing the percentiles of the normal range and calculating standard deviation scores (SDS) [SDS = $(x–X)$/SD; $x$ = IGFBP measured value in μg $l^{-1}$, $X$ = mean of IGFBP values in μg $l^{-1}$, which are characteristic of the age and sex of the single child; SD, standard deviation]. The significance of differences between groups was tested using Student's $t$-test ($P = 0.05$).

### RESULTS

### Serum level of IGFBP-1, -2 and -3

IGFBP-1, -2 and -3 concentrations were analysed in the serum from healthy children and diabetic children in comparison to ALL patients. The values obtained from controls were found to be between 12 and 177 μg $l^{-1}$ (IGFBP-1), 57 and 841 μg $l^{-1}$ (IGFBP-2) and 1331 and 4545 μg $l^{-1}$ (IGFBP-3).

**Table 1** Serum concentrations and SDS values from IGFBP-1, -2, -3 and IGF-I in healthy children and children at their initial presentation of diabetes

| | Healthy children | | | Diabetic children | | |
|---|---|---|---|---|---|---|
| | IGFBP-1 | IGFBP-2 | IGFBP-3 | IGFBP-1 | IGFBP-2 | IGFBP-3 |
| Concentration in (μg $l^{-1}$) | 12–53 | 54–468 | 1927–4414 | 15–177 | 90–706 | 1331–4813 |
| SDS range | −1.66–0.91 | −4.51–1.11 | −1.1–2.12 | −0.46–3.49 | −4.72–2.54 | -3.6–1.09 |
| SDS mean | −0.7 | −1.38 | 0.23 | 0.6 | −0.8 | −0.33 |

© Cancer Research Campaign 1998

**Figure 2** The detection of IGFBP-2 mRNA by RT-PCR. Lanes 1–10 document PCR fragments obtained from patients or control probands as marked, lane M represents the 100-bp marker (Gibco). The 496-bp DNA fragment is derived from the IGFBP-2 transcript, the 260-bp fragment represents the β-actin mRNA

Because of the age-dependent variations in IGFBP concentrations, the data are given in SDS. The normal range, represented by SDS from –2 to +2, includes 80% of all values from both control groups, the healthy probands as well as the diabetic children (Figure 1). Healthy children and children at their initial presentation of diabetes demonstrated averages of SDS of –0.7 and 0.6 (IGFBP-1), –1.38 and –0.8 (IGFBP-2), and 0.23 and –0.33 (IGFBP-3) respectively (Table 1 and Figure 1). The differences between both groups were found to be significant in IGFBP-1 ($P < 0.05$) but not in IGFBP-2 and -3. With respect to IGFBP-2 and -3, both groups were indistinguishable and therefore could be considered as a unique control population.

On the contrary, IGFBP-2 serum levels were found to be significantly elevated at the time of diagnosis in patients with ALL (Figure 1). The absolute concentrations of these factors were determined to be in the range of 9–148 µg $l^{-1}$ for IGFBP-1, 247–4048 µg $l^{-1}$ for IGFBP-2 or 433–4500 µg $l^{-1}$ for IGFBP-3. The averages of SDS of ALL patients were calculated to be 0.7 for IGFBP-1, 3.1 for IGFBP-2 or –1.7 for IGFBP-3 (Figure 1).

Significant differences ($P < 0.05$) were found for the IGFBP-2 values between the patients and the total control group as well as both control groups separately.

During the therapy, IGFBP-2 serum levels normalized. The absolute concentrations of IGFBP-2 determined at day 33 after the onset of chemotherapy was in the range of 64–905 µg $l^{-1}$ or represented an average SDS of –1.45. Only 2 of 14 samples (14%) investigated revealed elevated serum levels at this time (Figure 4).

### IGFBP-2 gene expression

To clarify whether the increased IGFBP-2 levels result at least partly from the tumour cells, we studied IGFBP-2 gene expression from leukaemic cells using RT-PCR. Using IGFBP-2-specific primers, we detected a 496-bp DNA fragment, a size that was expected from the known cDNA sequence (accession no. X16302) (Figure 2). To exclude false-negative results, we also investigated the occurrence of β-actin mRNA. This transcript was found in 97% (101 of 104) of all samples, indicating integrity of the RNA samples. The specificity of PCR products was confirmed by restriction analysis using the unique PpUM I site in the IGFBP-2 cDNA (Figure 3).

The IGFBP-2 transcript could be detected in 28% or 33% of MNC fractions derived from healthy children or children with diabetes respectively (Table 2). In children suffering from leukaemia, the situation was markedly altered. Sixty per cent of all patients displayed elevated IGFBP-2 serum levels at the time of diagnosis. The IGFBP-2 mRNA was detectable in nearly 72% of the corresponding MNC fractions of all patients (Table 2 and Figure 4). Calculating separately the detection rate of patients displaying increased or normal serum levels, the values were estimated to be 86% or 45% respectively (Figure 4).

© Cancer Research Campaign 1998

**Figure 3** Restriction analysis of IGFBP-2-derived PCR products. Five independent PCR fragments obtained from ALL patients were cut using PpUM I as described. In accordance with the cDNA sequence (X16302), two fragments with a length of 280 and 215 bp were detected. Lane 6 represents the undigested 496-bp PCR product

The observed normalization of the IGFBP-2 serum during chemotherapy was also accompanied by a decreased detection rate of the transcript. IGFBP-2 mRNA was detected in 35% of these samples only, a value similar to that determined for both control groups (Table 2 and Figure 4).

### DISCUSSION

It is known that malignant diseases lead to alterations in the IGF signalling system (Bergmann et al, 1995; Figueroa et al, 1995; Singh et al, 1996). Additionally, IGFs have been implicated in the pathology of numerous tumours (Daughaday and Deuel, 1991).

Recently, we reported on changes in IGF-I and -II and some of their binding proteins in children suffering from ALL and found the first hints that the increased IGFBP-2 serum level may correlate with the risk of relapse (Mohnike et al, 1996, 1997). As a result of the observation that the elevated IGFBP-2 levels generally normalized during chemotherapy, we concluded that the tumour clone may be directly or indirectly involved. In order to clarify the question whether tumour clones express IGFBP-2, we studied the corresponding gene expression of enriched tumour cells using RT-PCR and analysed these data with respect to the IGFBP-2 serum levels.

At the time of diagnosis, IGFBP-2-mRNA was detected in 72% of MNC samples isolated from ALL patients. In contrast, only 35% of the corresponding samples contained the IGFBP-2 transcript at day 33 of chemotherapy. This value is similar to that of healthy or diabetic children, estimated to be 28% or 33% respectively. The diabetic children were selected as a control group to exclude the possibility that elevated IGFBP-2 serum level in ALL patients simply originates from metabolic changes. Diabetic patients suffer from a well-characterized disease in which only insulin is lacking; they are comparable to ALL patients with respect to their age and their catabolic situation, which is caused by either insulin deficiency in diabetic children or tumour cachexia in ALL children. The metabolism of both groups is subjected to a caloric restriction. Both the diabetes control group and the ALL patients at time of diagnosis showed decreased IGF-I SDS values (Mohnike et al., 1996). As both the IGFBP-2 serum levels and the detection rate of the transcript are nearly identical in the healthy and diabetic children, metabolic alterations could not account for elevations of serum IGFBP-2 found in children suffering from ALL.

Furthermore, the results demonstrate that the majority of samples with increased IGFBP-2 serum levels was accompanied by the occurrence of IGFBP-2 mRNA in the corresponding MNC

**Figure 4** Correlation between the IGFBP-2 serum concentration and the occurrence of IGFBP-2 transcript in the corresponding MNC fraction. The dark area represents the normal distribution of IGFBP-2 serum levels given in SDS from 50 ALL patients at time of diagnosis and 14 ALL patients during remission (**A**) as well as from 15 diabetic children and 25 healthy probands (**B**). Each asterisk represents data obtained from one proband

 © Cancer Research Campaign 1998

**Table 2** IGFBP-2 serum levels and IGFBP-2 mRNA expression in children suffering from ALL, healthy controls and children with diabetes

| | IGFBP-2 mRNA | | IGFBP-2 serum level | |
|---|---|---|---|---|
| | Detectable | Not detectable | Elevated (above 2 SDS) | In normal range (between −2 and +2 SDS) |
| ALL patients at time of diagnosis % (*n*) | 72 (36) | 28 (14) | 60 (30) | 40 (20) |
| ALL patients in remission % (*n*) | 35 (5) | 65 (9) | 14 (2) | 86 (12) |
| Healthy children % (*n*) | 28 (7) | 72 (18) | 0 (0) | 100 (25) |
| Children at their initial presentation of diabetes % (*n*) | 33 (5) | 67 (10) | 20 (3) | 80 (12) |

fraction. This implies that changes in IGFBP-2 serum level could depend on the IGFBP-2 gene expression by the tumour clone. In addition, this assumption is supported by the adjustment of serum level and mRNA expression values observed after the destruction of the tumour clone by chemotherapy.

Taking together the data from control samples as well as patients during remission, it has to be noted that IGFBP-2 serum levels were detectable in all samples, however the presence of corresponding mRNA was shown in only 28–35%. Based on these data, it is obvious that IGFBP-2 levels result from other non-haematopoietic sources, e.g. liver. Therefore, serum IGFBP-2 has to be considered as a joint pool resulting from the gene expression of different cells. This assumption of other IGFBP-2 sources is in line with results of other authors demonstrating that haematopoietic cells contribute to the serum concentrations of the IGF-regulatory peptides (Shimon and Shipilberg, 1995).

Taking into account results from all 104 measurements, elevated serum levels but no mRNA were detected in five cases, four ALL patients and one diabetic child, whereas the serum IGFBP-2 was missing in six samples with positive mRNA values. At first glance, these results may be considered to be wrong and not in line with the hypothesis that the tumour clones represent the major cause of the elevated IGFBP-2 levels. However, the proportion of these data is less than 7% and they may reflect individual constitutions of the probands as well as the quality of samples. It is well known that an injury can lead to an increase in IGFBPs or IGFs caused by secretion by the liver (Lang et al, 1996) and therefore to an increase of serum IGFBP-2 without any cooperation of mononuclear cells. Otherwise, the technical limitations of the RIA or RT-PCR methods as well as differences during the transport, e.g. RNA degradation, could be regarded as possible explanations. However, despite the difficulties of explaining the exact cause of these results discussed above, the clear association between the occurrence of tumour cells and both parameters, the serum level and mRNA data have been proven. Until now, we were not able to definitely exclude the involvement of other tissues, such as the liver or epithelial cells, whose IGFBP-2 expression may be increased by the occurrence of tumour cells and therefore may cause elevated serum levels. However, the strong association of IGFBP-2 serum levels and the evidence of corresponding mRNA of the enriched tumour cells points to the conclusion that the elevated IGFBP-2 serum levels result from the gene expression of the tumour cells.

This finding is in accordance with other reports describing elevated IGFBP-2 levels in various malignant diseases, such as prostate cancer (Kanety et al, 1993), Wilms' tumour (Zumkeller et al, 1993) and lung cancer (Reeve and Payne, 1990). Whereas the stimulation of cell proliferation by IGFs is well established (Camacho-Hubner et al, 1991; Jones and Clemmons, 1995) the functions of IGFBP-2 on tumour and normal cells are still unknown. At the first sight, an increase in the IGF-binding proteins should lead to an inhibition of cellular proliferation by capturing IGFs. Therefore, the overexpression of IGFBP-2 seems to be in direct conflict with the explanation of the higher proliferation rate of lymphoblastic cells. One possible explanation may be that increased IGFBP-2 level serves as a storage pool of IGFs in the pericellular microenvironment of these cells. Although there is, as yet, no clear evidence, there are data supporting this idea. Roghani and co-workers reported that IGFBP-2 binds IGF-II rather than IGF-I (Roghani, 1991). Recently, it was shown that the IGF-II/IGFBP-2 complex is partly bound to the extracellular matrix (ECM) (Arai et al, 1996), where the IGF-II may be liberated from this complex by limited proteolysis. Gockerman and Clemmons (1995) described in porcine aortic smooth muscle cells a constitutively secreted serine protease capable of degrading IGFBP-2. Furthermore, they showed that the cleavage of IGFBP-2 into two fragments led to a strong reduction of the IGF-binding capacity, resulting in liberation of the IGF-II and subsequently to increased signal transduction (Binoux et al, 1994). Therefore, the increased IGFBP-2 levels may provide a storage pool of ECM-bound IGF/IGFBP-2 complex in the vicinity of tumour cells. Based on this hypothetical model, the IGFBP-2 could be considered to be advantageous for the tumour cells. However, it needs further experimental studies to confirm this hypothesis and reveal the functional role of IGFBP-2 tumour cells.

Taken together, the data presented here clearly provide evidence that the tumour clone itself is the major cause for the elevated IGFBP-2 serum levels in patients suffering from leukaemia and that the involvement of its gene expression may be the best explanation. This finding may be helpful for further studies aimed on the functional role of this binding protein and its potential as a marker and/or target for clinical studies.

### ACKNOWLEDGEMENTS

The authors thank Inga Handke and Bianka Hohmann for their valuable technical assistance. This work was supported by 'Deutsche Leukämie Forschungshilfe e.V.', grant no. 95.01 and 'Magdeburger Förderkreis krebskranker Kinder e.V.'

### REFERENCES

Adamo ML, Shao ZM, Lanau F, Chen JC, Clemmons DR, Roberts CTJ, Le Roith D and Fontana JA (1992) Insulin-like growth factor I (IGF-I) and retinoic acid modulation of IGF-binding proteins (IGFBPs): IGFBP-2, -3, and -4 gene expression and protein secretion in a breast cancer cell line. *Endocrinology* **131**: 1858–1866

Arai T, Busby W and Clemmons DR (1996) Binding of insulin-like growth factor (IGF) I or II to IGF-binding protein-2 enables it to bind to heparin and extracellular matrix. *Endocrinology* **137**: 4571–4575

© Cancer Research Campaign 1998

Bergmann U, Funatomi H, Yokoyama M, Beger HG and Korc M (1995) Insulin-like growth factor I overexpression in human pancreatic cancer: evidence for autocrine and paracrine roles. *Cancer Res* **55**: 2007–2011

Binoux M, Lalou C, Lassarre C and Segovia B (1994) Regulation of IGF bioavailability IGFBP proteases. In *The Insulin-like Growth Factors and their Regulatory Proteins, Proceedings of the Third International Symposium on IGFs*. Baxter RC, Gluckman PD and Rosenfeld RG. (eds), pp. 217–226 Elsevies: Amsterdam

Blum WF, Ranke MB, Kietzmann K, Gauggel E, Zeisel HJ and Bierich JA (1990) A specific radioimmunoassay for the growth hormone (GH)-dependent somatomedin-binding protein: its use for diagnosis of GH deficiency. *J Clin Endocrinol Metab* **70**: 1292–1298

Camacho-Hubner C, McCusker RH and Clemmons DR (1991) Secretion and biological actions of insulin-like growth factor binding proteins in two human tumor-derived cell lines in vitro. *J Cell Physiol* **148**: 281–289

Chard T, Blum WF, Brunjes J, Campbell DJ and Wathen NC (1994) Levels of insulin-like growth factor-binding protein-2 and insulin-like growth factor-II in maternal serum, amniotic fluid, and extraembryonic coelomic fluid at 9–20 weeks of pregnancy. *J Endocrinol* **142**: 379–383

Christofori G, Naik P and Hanahan D (1994) A second signal supplies by insulin-like growth factors II in oncogene-induced tumorgenesis. *Nature* **369**: 414–417

Clemmons DR (1991) Insulin-like growth factor binding proteins: roles in regulating IGF physiology. *J Dev Physiol* **15**: 105–110

Clemmons DR, Busby WH, Arai T, Nam TJ, Clarke JB, Jones JI and Ankrapp DK (1995) Role of insulin-like growth factor (IGF) binding proteins in the control of IGF actions. *Prog Growth Factor Res* **6**: 357–366

Czech MP (1989) Signal transmission by the insulin-like growth factors. *Cell* **59**: 235

Daughaday WH and Deuel TF (1991) Tumor secretion of growth factors. *Endocrinol Metab Clin North Am* **20**: 539–563

El-Brady OM, Romanus JA, Helman LJ, Cooper MJ, Rechler MM and Israel MA (1989) Autonomous growth of a human neuroblastoma cell line is mediated by insulin-like growth factor II. *J Clin Invest* **84**: 829–839

Figueroa JA, Lee AV, Jackson JG and Yee D (1995) Proliferation of cultured human prostate cancer cells is inhibited by insulin-like growth factor (IGF) binding protein-1: evidence for an autocrine growth loop. *J Clin Endocrinol Metab* **80**: 3476–3482

Gockerman A and Clemmons DR (1995) Porcine aortic smooth muscle cells secrete a serine protease for insulin-like growth factor binding protein-2. *Circ Res* **76**: 514–521

Jones J and Clemmons DR (1995) Insulin-like growth factors and their binding proteins: biological actions. *Endocr Rev* **16**: 3–34

Kanety H, Madjar Y, Dagan Y, Levi J, Papa MZ, Pariente C, Goldwasser B and Karasik A (1993) Serum insulin-like growth factor-binding protein-2 (IGFBP-2) is increased and IGFBP-3 is decreased in patients with prostate cancer: correlation with serum prostate-specific antigen. *J Clin Endocrinol Metab* **77**: 229–233

Kim H, Nagalla SR, Oh Y, Wilson E, Roberts CT and Rosenfeld RG (1997) Identification of a family of low affinity insulin-like growth factor binding proteins (IGFBPs): characterisation of connective tissue growth factor (CTGF) as a member of IGFBP superfamily. *Proc Natl Acad Sci USA* **94**: 12981–12986

Lang CH, Fan J, Frost RA, Gelato MC, Sakurai Y, Herndon DN and Wolfe RR (1996) Regulation of the insulin-like growth factor system by insulin in burn patients. *J Clin Endocrin Metab* **81**: 2427–2480

Li YM, Arkins S, Rebeiz N and Kelley KW (1993) Autocrine regulation of human myeloid cell lines by insulin-like growth factor-I (IGF-I), IGF binding proteins (IGFBP) and the type I IGF receptor (IGF-IR). *J Immunol* **150**: 53

Lino K, Seppala M, Heinonen PK, Sipponen P and Rutanen EM (1986) Elevated levels of a somatomedin-binding protein PP12 in patients with ovarian cancer. *Cancer* **58**: 2294–2297

Martin DM, Singleton JR, Meghani MA and Feldman EL (1993) IGF receptor function and regulation in autocrine human neuroblastoma cell growth. *Regul Peptides* **48**: 225–232

Melino G, Knight RA and Thiele CJ (1993) New insight on the biology of neuroectodermal tumours. *Cancer Res* **43**: 926–928

Mohnike K, Kluba U, Mittler U, Aumann V, Vorwerk P and Blum W (1996) Serum levels of insulin-like growth factor-I, -II and insulin-like growth factor binding proteins -2 and -3 in children with acute lymphoblastic leukaemia. *Eur J Pediatr* **155**: 81–86

Mohnike K, Wex H, Vorwerk P, Kluba U, Aumann V, Mittler U and Blum W (1997) High serum IGFBP-2 in acute lymphoblastic leukaemia (ALL) may be an indication for an increased risk of relapse. In *Acute Leukemias VII: Prognostic Factors and Treatment Strategies*, Büchner T. (ed.), Springer: Berlin (in press)

Neely EK, Beukers WM, Oh Y, Cohen P and Rosenfeld RG (1991) Insulin-like growth factor receptors. *Acta Pediatr Scand* **372** (suppl.): 116–123

Rechler MM (1993) Insulin-like growth factor binding protein. *Vitam Horm* **47**: 1–114

Reeve JG and Payne JA (1990) Production of immunoreactive insulin-like growth factor I (IGF-I) and IGF I binding proteins by human lung tumours. *Br J Cancer* **61**: 727–731

Reeve JG and Kirby LB (1992) Insulin-like growth factor binding protein gene expression and protein production by human tumour cell lines. *Int J Cancer* **51**: 818–821

Reeve JG, Brinkman A, Hughes S, Michell J, Schwander J and Bleehen NM (1992) Expression of insulin-like growth factor (IGF) and IGF-binding protein genes in human lung tumor cell lines. *J Natl Cancer Inst* **84**: 628–634

Roghani M, Lassarre C, Zapf J, Povoa G and Binoux M (1991) Two insulin-like growth factor (IGF)-binding proteins are responsible for the selective affinity for IGF-II of cerebrospinal fluid binding proteins. *J Clin Endocrinol Metab* **73**: 658–666

Shimon I and Shpilberg O (1995) The insulin-like growth factor system in regulation of normal and malignant hematopoesis. *Leuk Res* **19**: 233–240

Singh P, Dai BS, Yallampalli U, Lu XB and Schroy PC (1996) Proliferation and differentiation of a human colon cancer cell line (CaCo2) is associated with significant changes in the expression and secretion of insulin-like growth factor (IGF) IGF-II and IGF-binding protein-4: role of IGF-II. *Endocrinology* **137**: 1764–1774

Zumkeller W, Schwander J, Mitchell, CD, Morrell, DJ, Schofield, PN and Preece MA (1993) Insulin-like growth factor (IGF)-I, -II and IGF-binding protein-2 (IGFBP-2) in the plasma of children with Wilms tumour. *Eur J Cancer* **29**: 1973–1977

© Cancer Research Campaign 1998